# Emotional valence through pupil: Machine learning classification under controlled visual complexity and emotional arousal in young adults

**DOI:** 10.14814/phy2.70793

**Published:** 2026-03-03

**Authors:** Jung Joo Lee, Eun Seo Park, Hwa Jin Han, Young Il Cho

**Affiliations:** ^1^ College of Police Administration Dongguk University Seoul Republic of Korea; ^2^ Division of Police Administration Dongguk University Seoul Republic of Korea

**Keywords:** emotional arousal, emotional valence, machine learning, pupillometry, spatial frequency

## Abstract

Pupillometry has long been proposed as a noninvasive physiological measure for emotional valence. However, its empirical effectiveness remains inconclusive due to confounding visual and emotional factors. This study examined whether pupil response patterns alone can reliably distinguish between positive and negative emotional stimuli while explicitly controlling for visual complexity (spatial frequency; SF) and emotional arousal at three standardized levels. Fifty images (25 positive and 25 negative) were presented, and pupil responses were recorded. Dynamic time warping‐based clustering captured temporal variations and similarities in pupil size responses across visual conditions. Initial classification without controlling SF and arousal yielded near‐chance accuracy (~57%) despite luminance control. However, performance improved substantially when stimuli were segmented by specific arousal–SF combinations. Under a representative low arousal, high spatial‐frequency condition (SF level 4), the best‐performing configuration (logistic regression) achieved a mean classification accuracy of approximately 79% and an AUC of 0.88, with consistently high precision, recall, and specificity across cross‐validation folds. Feature importance analyses highlighted critical pupillary parameters, including the area under the pupil dilation curve, as key predictors. These results suggest that pupillary responses can reliably indicate emotional valence under rigorously controlled visual conditions, emphasizing control of perceptual and emotional factors in pupillometry‐based emotion research.

## 1 INTRODUCTION

Given the significant implications that emotions have for behavior, understanding emotional states has long been a central focus in psychology and psychophysiology, cognition, and well‐being (Bradley & Lang, 2000). Pupillometry is a physiological technique for evaluating emotional processes by measuring changes in pupil diameter, reflecting underlying neural and emotional activity (Beatty, 1982). Historically, larger pupils have been associated culturally and scientifically with more positive emotional states, which was first empirically supported by Hess and Polt (1960). However, subsequent research aiming to replicate and validate these initial findings has yielded inconsistent and often inconclusive results, leading researchers to question the reliability of pupil dilation as a marker of emotional valence (Partala & Surakka, 2003).

Several factors may account for these inconsistencies, notably the influence of visual complexity—often operationalized as spatial frequency—and emotional arousal levels inherent in experimental stimuli (Bradley et al., 2008). Both visual complexity and arousal significantly impact cognitive and neural processing, which, in turn, affect physiological responses such as pupil dilation (Huang & Clewett, 2024). Despite acknowledgment of these confounding factors, few previous studies have controlled for them, potentially obscuring genuine valence‐related pupil responses (Hershman et al., 2024; Partala & Surakka, 2003).

In addition to these stimulus‐driven factors, pupil dilation is also modulated by higher‐order cognitive processes such as attentional allocation, task demands, and cognitive load. Classic work has demonstrated that task‐evoked pupillary responses systematically increase with processing demands, reflecting the allocation of limited cognitive resources rather than emotional arousal per se (Beatty, 1982; Kahneman, 1973). These cognitive influences have been consistently observed across experimental and clinical contexts (Granholm et al., 1996) and may act as important confounding factors in studies examining emotion‐related pupillary responses.

This study addresses these methodological limitations by revisiting the potential for pupillary dynamics to reliably distinguish between positive and negative emotional valence, explicitly controlling for both spatial frequency and emotional arousal. In doing so, it aims not only to clarify previous conflicting findings but also to provide a more nuanced understanding of the relation between emotional valence and pupillary response. Clarifying the relation holds considerable theoretical significance for psychophysiology and cognitive neuroscience and practical relevance for clinical diagnostics and emotion‐sensitive technologies.

### 1.1 Pupillometry and emotion

Pupillometry, the measurement of variations in pupil diameter, has emerged as a valuable noninvasive physiological tool in psychological and neuroscientific research (Bradley et al., 2008; Hess, 1965; Hess & Polt, 1960; Loewenfeld, 1993). Changes in pupil size are primarily regulated by the autonomic nervous system through the dynamic balance between sympathetic and parasympathetic activity (Bradley et al., 2008; Loewenfeld, 1993). In general, sympathetic activation is associated with pupil dilation, whereas parasympathetic activation is linked to pupil constriction; however, the expression of these mechanisms in emotional contexts is influenced by multiple interacting factors, including stimulus properties and task demands (Bradley et al., 2008).

Pupil responses have been associated with neural activity in brain regions involved in arousal regulation and emotional processing, such as the hypothalamus, locus coeruleus, and amygdala (Costa & Rudebeck, 2016; Huang & Clewett, 2024). Emotional experiences are commonly characterized along two core dimensions: valence, referring to the positive or negative quality of an emotion, and arousal, reflecting its intensity. Both dimensions can influence autonomic activity and, consequently, pupillary responses (Bradley et al., 2008; Cash et al., 2024; Partala & Surakka, 2003).

Early work suggested that pupil dilation may be more pronounced for positive emotional stimuli, leading to the hypothesis that pupillary responses could serve as a marker of emotional valence (Hess, 1965). However, subsequent research attempting to replicate these findings has produced inconsistent results. Many studies have demonstrated that pupil size is more reliably modulated by emotional arousal and cognitive demands than by emotional valence alone (Bradley et al., 2008; Partala & Surakka, 2003). Negative emotional stimuli, in particular, have been reported to elicit both dilation and constriction depending on contextual factors, including stimulus modality, arousal level, and experimental task, rather than showing a uniform or valence‐specific pattern.

More recent empirical work has further challenged the notion of a fixed mapping between emotional valence and pupillary direction. Across visual and auditory domains, contemporary studies consistently report pupil dilation in response to both positive and negative emotional stimuli, with the magnitude and temporal dynamics of the response being shaped primarily by arousal, stimulus complexity, and attentional engagement (Babiker et al., 2015; Kirwan et al., 2025; Oliva & Anikin, 2018; Snowden et al., 2016). These findings suggest that pupillary responses to emotion are better understood as reflecting dynamic interactions between affective and cognitive processes rather than a simple index of emotional valence.

In this context, the present study does not assume a one‐to‐one correspondence between negative emotional valence and pupillary constriction. Instead, it focuses on relative differences in pupil size dynamics between positive and negative conditions under tightly controlled stimulus properties, allowing for a more nuanced examination of valence‐related effects within the broader and still‐evolving literature on emotional modulation of pupil responses.

### 1.2 Visual characteristics and pupil responses

Aside from emotional influences, pupil size is significantly affected by specific visual characteristics of stimuli, such as luminance and spatial frequency (Bradley et al., 2008; Kim et al., 2023; Loewenfeld, 1993). Luminance, defined as the brightness of an image, is a primary determinant of pupil diameter, with brighter images typically inducing pupil constriction (Bradley et al., 2008; Loewenfeld, 1993).

Spatial frequency, referring to the rate of luminance changes within an image, also influences pupil responses (Delplanque et al., 2007; Kim et al., 2023). Intermediate spatial frequencies (approximately 2–8 cycles per degree) have been found to induce greater pupil constriction, likely due to increased cognitive processing demands associated with visual detail perception and attentional focus (Hu et al., 2019).

Previous research attempting to use pupillary responses to distinguish emotional valence appears to have insufficiently controlled for potentially confounding variables such as luminance, spatial frequency, and emotional arousal (Bradley et al., 2008; Delplanque et al., 2007; Kim et al., 2023). To clarify the debated relation between pupil responses and emotional valence, the current study explicitly controlled for these visual and arousal variables. By carefully managing these factors, it aims to more precisely assess pupil diameter as a potential indicator of emotional valence.

### 1.3 Machine learning

Recent studies have actively integrated physiological data, such as pupil measurements, with various machine learning techniques (Islam & Bae, 2024; Jiang et al., 2024; Mohamed et al., 2024). Binary classification, a machine learning task aimed at categorizing data into two distinct groups, is frequently utilized in physiological research (Breiman, 2001; He & Garcia, 2009). Typically, machine learning models predict category membership based on features extracted from physiological signals. However, physiological datasets often contain many features relative to the number of available samples, posing a risk of overfitting due to high dimensionality (He & Garcia, 2009). Therefore, careful selection of classifiers and rigorous evaluation methods, such as cross‐validation, are essential to enhance the model's generalizability.

Dynamic time warping (DTW) is an effective method for analyzing similarities in time series data. DTW measures similarity between sequences that differ in length or timing, making it especially useful for accurately capturing temporal patterns in physiological signals (Kang et al., 2014). In this study, DTW clustering was employed to group pupil diameter time series data. DTW clustering was used solely for exploratory analysis of temporal similarity patterns and was not used for feature extraction or classifier training.

Class imbalance can significantly impair model performance and reduce predictive accuracy for minority classes. Various oversampling techniques have been developed to address this issue, with the synthetic minority over‐sampling technique (SMOTE; Chawla et al., 2002) widely acknowledged as an effective method for synthesizing additional minority‐class samples to achieve class balance. Although this study initially balanced positive and negative images (25 each), controlling for emotional arousal levels and spatial frequency levels could potentially lead to imbalanced subsets. To mitigate such an imbalance, SMOTE oversampling was applied.

Cross‐validation is commonly employed in machine learning to evaluate model performance. When data are grouped (e.g., by participants), group k‐fold cross‐validation effectively prevents data leakage and objectively assesses model generalizability (Liu et al., 2024; Roberts et al., 2017). Given that this study structured data as participant‐by‐stimulus combinations, group k‐fold cross‐validation was used, grouping data at the participant level.

Traditionally, machine learning models have been viewed as “black boxes,” providing output without clear explanations regarding the direction or magnitude of each feature's contribution. Recently, methods such as SHapley Additive exPlanations (SHAP; Lundberg & Lee, 2017) have improved interpretability by clarifying feature importance and directionality (positive or negative contributions). SHAP analyses clearly indicate how each feature influences model predictions, making this approach particularly beneficial for physiological data analysis (Liu et al., 2022; Scapin et al., 2022). This study employed SHAP analyses to assess feature contributions explicitly.

## 2 METHODS

### 2.1 Participants and experimental procedure

Participants were recruited via on‐campus advertisements and voluntarily agreed to take part in the experiment. Written informed consent was obtained from all participants before participation, including consent for data collection and the use of personal information for research purposes. The study was approved by the Institutional Review Board (IRB). Detailed information, including the IRB approval number, is reported in the Ethics Statements.

A total of 40 undergraduate students participated in the study (20 males and 20 females). Participants ranged in age from 20 to 30 years (M = 22.75, SD = 2.51). All participants reported normal or corrected‐to‐normal vision, and none reported a history of neurological or psychiatric disorders.

The experimental task involved passive viewing of visual stimuli. Participants were instructed to maintain their gaze on the center of the screen and to observe each image as it was presented, without making any overt responses.

The stimulus set consisted of 50 grayscale images, including 25 positive and 25 negative stimuli. All images were originally purchased from Shutterstock and subsequently converted to grayscale. Brightness and luminance were normalized across images to reduce low‐level visual confounds. Due to copyright restrictions and licensing terms, the original images cannot be distributed as part of this publication. Consequently, the use of commercially sourced images may limit the full reproducibility of the stimulus set, a limitation acknowledged in the present study.

Emotional valence labels (positive or negative) were adopted from the original categorizations provided by Shutterstock. The criteria used to define emotional arousal levels and spatial frequency (SF) categories were based on conceptual criteria derived from the International Affective Picture System (IAPS). Images were categorized into low, medium, or high arousal, as well as into five SF levels, according to their semantic, affective, and visual characteristics. Detailed descriptions of the arousal and SF categorization criteria, along with representative image characteristics, are reported in Section 2.5 (Emotional Arousal and Spatial Frequency) and summarized in Table 4.

To ensure transparency, raw stimulus properties were explicitly reported. Each image was characterized in terms of its original valence category (positive or negative), arousal category, spatial frequency characteristics, and mean luminance values after luminance normalization. Descriptive statistics and stimulus‐level values for these properties are provided in Table 1.

**TABLE 1:** Descriptive statistics of stimulus properties.

Property	Category/Scale	Value
Valence	Positive	25
Valence	Negative	25
Arousal rating	1–7 scale	4.62 ± 1.64
Arousal level	Low (1–2)	7
Arousal level	Medium (3–5)	25
Arousal level	High (6–7)	18
Spatial frequency	Continuous	14.27 ± 8.61
SF level	1 (5.40–6.95)	7
SF level	2 (7.19–11.06)	11
SF level	3 (11.08–13.30)	10
SF level	4 (13.32–17.68)	11
SF level	5 (17.93–47.82)	11
Mean luminance	Normalized	127.52 ± 0.07

*Note*: Arousal ratings were obtained on a 7‐point scale and subsequently categorized into low (1–2), medium (3–5), and high (6–7) arousal levels. Spatial frequency values represent continuous measures of image spatial complexity and were further grouped into five ordered SF levels with approximately balanced numbers of images per level. Mean luminance values reflect average pixel luminance after luminance normalization. Values are reported as mean ± standard deviation or counts, as appropriate.

Pupil measurements were recorded using a monocular (right‐eye) eye‐tracking system (Pupil Labs) with a sampling rate of 60 frames per second (fps). Before data collection, a standard camera calibration procedure was performed for each participant to ensure accurate pupil tracking. Recorded pupil sizes were normalized to an individual baseline of 100, and all values were expressed as relative ratios (D_RATIO).

The experiment was conducted in a darkened environment designed to minimize external visual interference. The recording area consisted of an enclosed structure approximately 2 meters in height, covered with black fabric. Participants were seated approximately 90 cm from the display monitor. Each stimulus was presented for 6 s (approximately 360 frames), followed by a 3‐s inter‐stimulus interval during which a uniform gray screen (RGB: 125, 125, 125) was displayed. Pupil measurements were paused during these gray‐screen intervals, which served as baseline adjustment periods. The total duration of the experimental session was approximately 10 min.

### 2.2 Data preprocessing

When processing pupillometric data, applying preprocessing that carefully considers physiological characteristics is essential (Hershman et al., 2024). Specifically, pupil data often contain noise and missing values, necessitating procedures such as handling abrupt frame‐to‐frame variations and interpolation to maintain signal reliability (Hershman et al., 2024). In this study, preprocessing of pupil‐response time series data was conducted according to the following criteria to ensure physiological validity and measurement reliability:

First, frames in which the change in D_RATIO exceeded 10% between consecutive frames (measured at 60fps) were considered as noise due to abrupt changes and, thus, were marked as missing values starting from that frame. The missing data interval ended when the change returned to within 5%. If these missing intervals spanned 30 frames or fewer, they were interpolated using Piecewise Cubic Hermite Interpolating Polynomial (PCHIP; Fritsch & Carlson, 1980). However, samples with missing intervals exceeding 30 frames were excluded from analysis.

Next, frames in each sample with D_RATIO values below 40 or above 160 were marked as missing. Similarly, missing intervals lasting 30 frames or fewer were interpolated using PCHIP, whereas those exceeding 30 frames led to exclusion of the sample from further analysis. This threshold of 30 frames corresponds to twice the minimum duration required (250 ms, approximately 15 frames at 60fps) for pupil size changes to reflect external stimuli or internal cognitive processes (Costa & Rudebeck, 2016; Kang et al., 2014).

Through this preprocessing procedure, among 2000 stimulus‐based responses (40 participants × 50 image stimuli), samples containing continuous frame‐level missing values or feature‐specific missing values were excluded, resulting in a final dataset of 1644 valid samples.

### 2.3 Feature extraction

**FIGURE 1:**
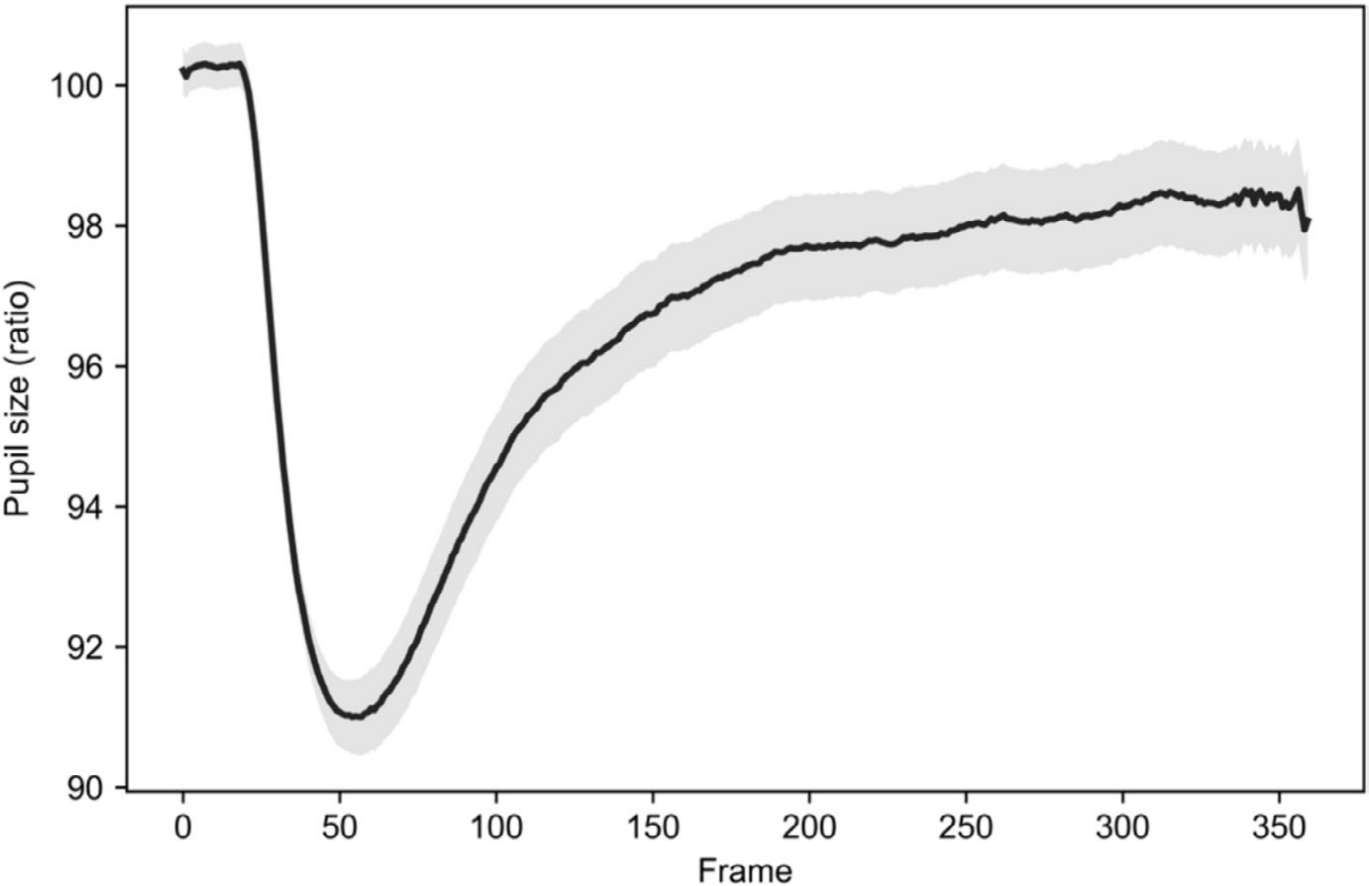
Overall average pupil response across stimuli. The solid line represents the grand‐average pupil size across all time series, and the shaded area indicates ±1 standard error of the mean (SEM). The figure illustrates the general temporal dynamics of pupil response during stimulus presentation.

The features used in this study were derived from visual exploration of the pupil response time‐series patterns presented in Figure 1. Images were categorized into two groups, positive and negative, and their corresponding pupil responses were visually inspected over time. The extracted features were designed to capture the distinctive pupil response pattern characterized by an initial constriction followed by a subsequent recovery phase, incorporating specific metrics such as drop ratio, recovery ratio, and the area under the pupil curve (AUPC). All features used for machine‐learning classification were extracted directly from the original preprocessed pupil time series.

In the graphs in Figure 1, positive stimuli are represented by solid lines, while negative stimuli are indicated by dotted lines. Additionally, differences were observed in pupil size ratios between the initial frame (frame 1) and the final frame (frame 360). Through visual waveform analysis, a total of 45 features were extracted, including basic statistical measures as well as derived metrics capturing cumulative changes across frames. The extracted pupil‐related features are presented in Table 2.

**TABLE 2:** Definition of pupil‐based features used in machine learning.

Feature	Definition
Max	Maximum pupil size
Min	Minimum pupil size
Mean	Mean pupil size
Std	Standard deviation of pupil size
Start	Pupil size at frame 1
Finish	Pupil size at frame 360
Peak	Maximum pupil size after frame 50
Peak ratio	Peak pupil size compared to minimum size
Peak latency	Time to reach peak pupil size
Drop latency	Time to reach minimum pupil size
Drop ratio	Minimum pupil size compared to initial pupil size
Recovery ratio	Final pupil size compared to initial pupil size
Response duration	Peak latency−drop latency
Reaction extent	Drop ratio + recovery ratio
Stability	Mean/std
Range	Max–min
Baseline deviation	Start−mean
AUPC	Area under pupil size curve (360 frames)
s AUPC	Area under pupil size curve after scaling start to 100 (360 frames)
AUPC1~6	AUC calculated in 6 intervals of 60 frames each
s AUPC1~6	Standardized AUC calculated in 6 intervals of 60 frames each
Change sum	Sum of absolute frame‐to‐frame changes (360 frames)
s change sum	Sum of absolute changes after scaling start to 100 (360 frames)
Change sum1~6	Change sum calculated in 6 intervals of 60 frames each
s change sum1~6	Standardized change sum calculated in 6 intervals of 60 frames each

*Note*: AUPC‐based features were designed to capture cumulative characteristics of pupil dynamics across the entire stimulus presentation period, complementing point‐based measures such as peak or minimum pupil size. Standardized variants (sAUPC and sAUPC1–6) were included to enable comparisons across trials by normalizing initial pupil size. Several of these cumulative metrics were specifically developed for the present study to capture temporal aspects of pupillary responses not represented by conventional summary statistics.

### 2.4 Classification model and validation

In this study, ten different classifiers were utilized to distinguish between positive versus negative groups. For example, logistic regression, support vector machine, decision tree, random forest, K‐nearest neighbors, XGBoost, LightGBM, easy ensemble, balanced random forest, and RUSBoost were employed. A fixed random state (seed = 1004) was consistently applied across all stages of the analysis, including group k‐fold cross‐validation splits and classifier training to ensure full reproducibility of the results.

Model performance was evaluated using group k‐fold cross‐validation. The group k‐fold method divides the dataset into k subsets (folds), ensuring that samples from the same participant do not simultaneously appear in both the training and validation sets, thus preventing data leakage (Peng et al., 2002). Classification performance was assessed based on six metrics: accuracy, precision, recall, F1 score (harmonic mean of precision and recall), specificity, and AUC. The performance metrics are presented in Table 3.

**TABLE 3:** Performance metrics used in the model evaluation.

Metric	Definition
Accuracy	The proportion of correctly classified cases out of all predictions made.
Precision	The proportion of true positive cases among those predicted as positive.
Recall	The proportion of actual positive cases correctly identified as positive.
F1 score	The harmonic mean of precision and recall balances the two metrics.
Specificity	The proportion of actual negative cases correctly identified as negative.
AUC	A comprehensive measure of the model's overall discriminatory ability.

To ensure fair comparison across classifiers and to avoid overfitting, given the limited sample size within certain arousal × spatial frequency subsets, no extensive hyperparameter optimization was performed. Instead, all classifiers were trained using standard or commonly adopted default configurations, with only essential parameters (e.g., number of neighbors for k‐NN, number of folds for cross‐validation) explicitly specified. All hyperparameter settings used in the analyses are summarized in Section 3.

However, for tasks requiring a single representative metric—such as heatmap or contour plot visualization—none of the individual metrics alone sufficiently captured the overall classification balance. Therefore, we additionally computed the P4 score, a composite performance metric defined as the harmonic mean of precision, recall, specificity, and negative predictive value (NPV). The P4 score was chosen to ensure a more conservative and balanced evaluation of model performance, as recommended in the balanced metric family framework (Marra, 2024).

### 2.5 Emotional arousal level and spatial frequency level

Emotional arousal was defined with reference to the conceptual framework of the International Affective Picture System (IAPS; Lang et al., 2005), which is based on a 9‐point arousal scale. However, images corresponding to the extreme ends of the scale (ratings of 8–9) were intentionally excluded from the stimulus set. Highly positive images in this range often contained sexually explicit content, while highly negative images frequently depicted graphic or violent scenes. Such content was deemed inappropriate for the present study due to ethical considerations and the potential for participant discomfort.

Accordingly, arousal ratings were operationalized within a restricted 1–7 range. Images were categorized into three arousal levels using predefined thresholds: ratings of 1–2 were classified as low arousal, ratings of 3–5 as medium arousal, and ratings of 6–7 as high arousal. Conceptual definitions and representative image characteristics for each arousal level are summarized in Table 4.

**TABLE 4:** Conceptual definitions and example image characteristics for Arousal Levels.

Arousal level	Example image characteristics
Low	Neutral, calm, peaceful scenes with minimal emotional engagement
Medium	Every day social scenes, moderate emotional expressions
High	Strongly engaging or stimulating scenes (e.g., conflict, fear, excitement, and intense joy)

*Note*: The table provides qualitative descriptions and representative image characteristics for low, medium, and high arousal categories used to guide stimulus classification. Quantitative arousal ratings, spatial frequency values, and descriptive statistics corresponding to these categories are reported in Table 1.

Spatial frequency (SF) was quantified for each image as a continuous measure of visual complexity. SF levels were defined by partitioning the continuous SF distribution into five ordered categories with approximately balanced numbers of images per level. The corresponding continuous SF ranges and the number of images assigned to each SF level are reported in Table 1, while conceptual descriptions of SF levels are provided in Table 4.

Importantly, emotional arousal level and spatial frequency were not used as input features to the machine‐learning classifiers. Instead, these variables were used exclusively to stratify the dataset into subset‐specific models, allowing model performance to be examined under different arousal and spatial frequency conditions.

Spatial frequency was computed using a gradient‐based measure that captures local intensity variations in the horizontal and vertical directions of the image. Mean luminance was calculated as the average pixel luminance of each grayscale image following luminance normalization.

### 2.6 SMOTE over‐sampling strategy and optimal case determination

In this study, the Synthetic Minority Over‐sampling Technique (SMOTE) was employed to address potential class imbalance arising during classification analyses that simultaneously controlled for emotional arousal and spatial frequency (SF). Although the original stimulus set was designed to be balanced with respect to emotional valence (25 positive and 25 negative images), an effective imbalance emerged after preprocessing due to participant‐specific data loss. In particular, blink‐related frame rejection, tracking failures, and other sources of signal noise resulted in unequal numbers of valid samples contributing to some arousal × SF × valence subsets.

When training classification models on imbalanced datasets, insufficient representation of minority‐class samples can lead to degraded predictive performance, particularly affecting recall‐ and F1‐related metrics. To mitigate these effects and to ensure stable and robust model training across subset‐specific analyses, SMOTE was adopted as a conservative oversampling strategy.

SMOTE generates synthetic samples by interpolating along line segments connecting existing minority‐class samples and their k‐nearest neighbors, with a commonly used default k‐value of 5 (Chawla et al., 2002). However, when the number of available minority‐class samples is limited—as was the case in certain arousal × SF subsets—using relatively large k‐values may introduce redundancy or excessive smoothing in the synthetic samples. To address this concern, we systematically explored a range of k‐values from 1 to 5, with particular emphasis on lower k‐values (k = 1 or 2), which allow more localized interpolation directly from original minority‐class samples.

Importantly, SMOTE was applied exclusively within the training folds of the group k‐fold cross‐validation procedure. Oversampling was never performed on the full dataset before partitioning. For each cross‐validation split, data from held‐out participants were completely excluded before oversampling, ensuring that synthetic samples were generated only from training participants and preventing any information leakage across subjects.

Based on this parameter exploration, the optimal oversampling condition was selected as the configuration yielding the highest P4 score. The P4 score provides a conservative and balanced summary of classification performance by integrating precision, recall, specificity, and negative predictive value, and was therefore used as the primary criterion for determining the optimal case reported in the main analyses.

The experimental procedure and participant details presented in this paper partially overlap with those described in another manuscript currently under review. The other manuscript employs the same methodological framework but investigates different research questions and analytical approaches.

## 3 RESULTS

### 3.1 Initial valence classification

In the initial classification attempts conducted without explicit control of emotional arousal or spatial frequency, overall classification performance remained modest and close to chance level. Across multiple machine‐learning models, mean accuracy values clustered in the mid‐50% range, with corresponding AUC values also remaining low (Table 5). These results indicate that, in the absence of systematic control over stimulus‐driven factors, pupillary response features alone provide limited discriminative power for emotional valence classification.

**TABLE 5:** Initial valence classification performance based on cross‐validation averages.

CV_folds	Model	Accuracy	Precision	Recall	Specificity	F1	AUC
5	LogisticRegression	0.571 ± 0.039	0.590 ± 0.032	0.763 ± 0.054	0.328 ± 0.102	0.664 ± 0.028	0.560 ± 0.050
5	BalancedRandomForest	0.554 ± 0.023	0.596 ± 0.022	0.616 ± 0.044	0.475 ± 0.028	0.606 ± 0.030	0.558 ± 0.030
7	LightGBM	0.540 ± 0.042	0.579 ± 0.042	0.638 ± 0.056	0.417 ± 0.060	0.606 ± 0.041	0.555 ± 0.053
7	EasyEnsemble	0.547 ± 0.042	0.601 ± 0.035	0.555 ± 0.104	0.539 ± 0.081	0.572 ± 0.064	0.554 ± 0.048
7	LogisticRegression	0.570 ± 0.038	0.585 ± 0.019	0.783 ± 0.104	0.305 ± 0.062	0.667 ± 0.046	0.554 ± 0.058

*Note*: The table shows the classification performance of multiple machine‐learning models without explicit control of emotional arousal or spatial frequency. Values represent (mean ± standard deviation) across group k‐fold cross‐validation folds.

Importantly, these findings should not be interpreted as evidence that pupillary responses lack valence‐related information. Rather, they underscore the central motivation of the present study: strong stimulus‐driven factors, such as emotional arousal and visual complexity, can dominate pupillary dynamics and obscure more subtle valence‐related signals when not explicitly controlled.

### 3.2 DTW‐based clustering results and impact of visual complexity

DTW‐based clustering was applied solely as an exploratory analysis to examine similarities in the temporal dynamics of pupil responses. The resulting clusters were used for descriptive interpretation and were not used as input features for any machine‐learning classifiers.

**FIGURE 2:**
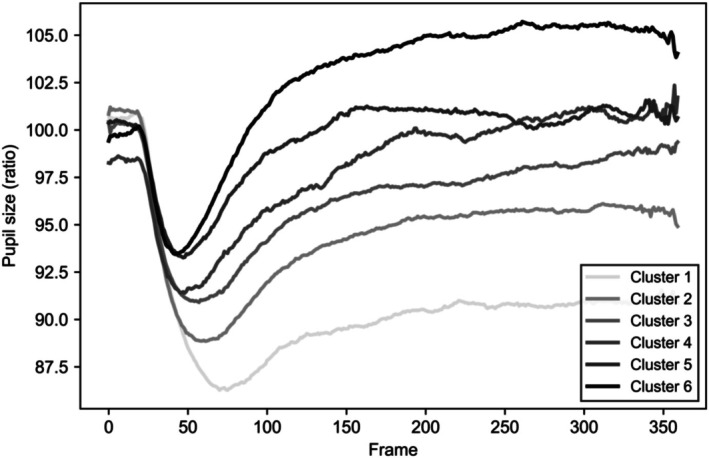
Average pupil response per cluster. Pupil response time series were clustered using DTW‐based k‐means (*k* = 6). Each line represents the average pupil response of a cluster. Line intensity reflects the relative magnitude of the cluster‐wise mean pupil size, ranging from lighter gray (smaller mean) to darker gray (larger mean). Clusters are numbered in ascending order of their mean pupil size.

Figure 2 illustrates the result of DTW clustering of images based on pupil size responses. Among the identified clusters, the most noteworthy are Cluster 1 and Cluster 6, representing the extreme patterns of pupil size dynamics. Specifically, Cluster 6 shows the largest overall pupil size, whereas Cluster 1 demonstrates the smallest pupil size throughout the measurement period.

**FIGURE 3:**
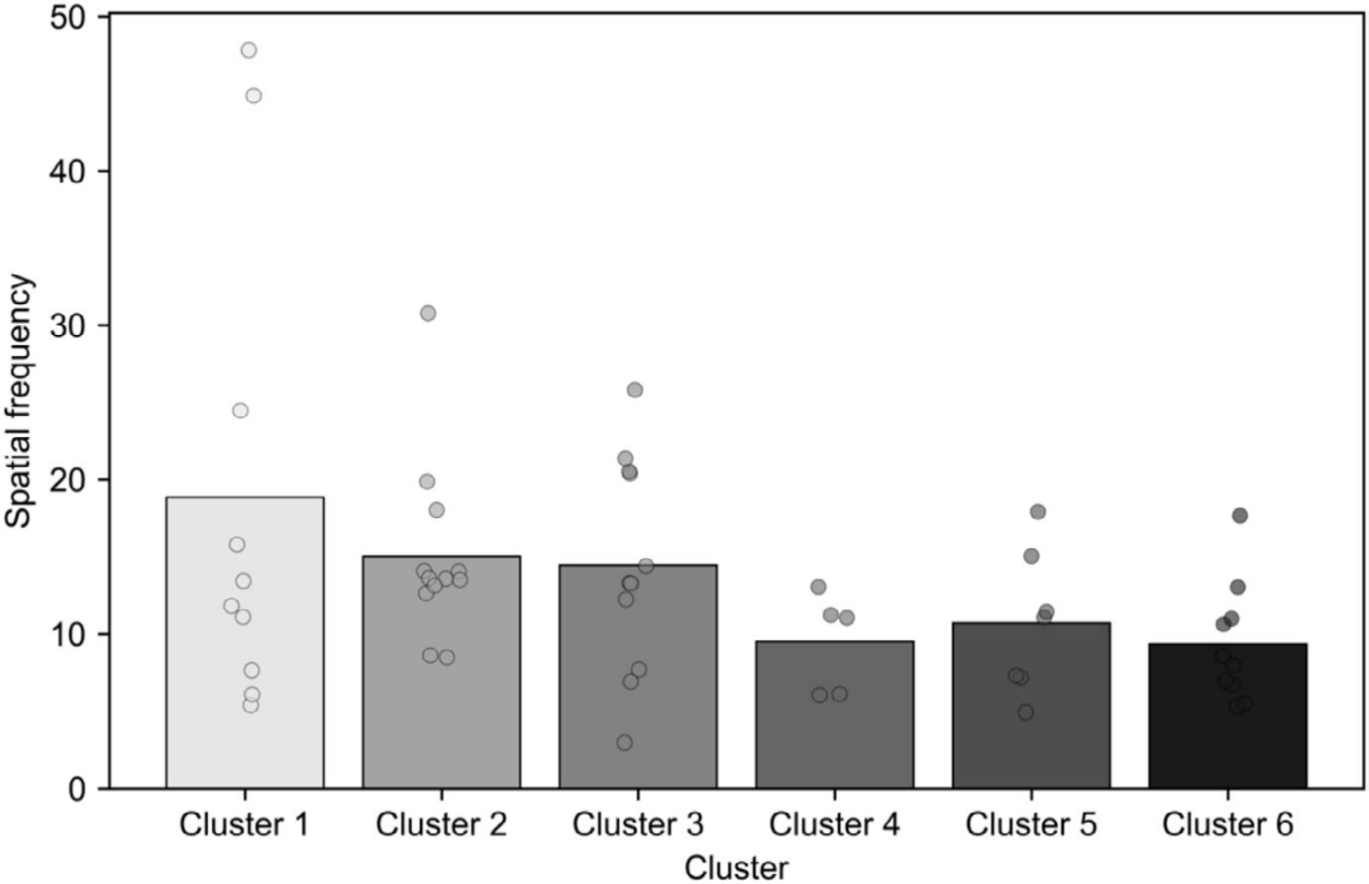
Mean spatial frequency by cluster. Bars represent the mean spatial frequency of stimuli assigned to each cluster, while individual data points indicate the spatial frequency of each stimulus within the cluster. Clusters correspond to those shown in Figure 2 and are ordered by increasing mean pupil size. The bar and point colors match the grayscale scheme used in Figure 2 and serve solely to identify clusters.

Figure 3 depicts the average spatial frequency values for each cluster. SF refers to the frequency of luminance changes per pixel, with higher values indicating more frequent and detailed changes in contrast. As illustrated, Cluster 6 corresponds to the lowest mean SF (9.35), while Cluster 1 exhibits the highest SF (18.85).

Returning to Figure 2, we observe that Cluster 1, which is associated with the highest spatial frequency, displays a rapid and pronounced pupil constriction around frame 50. Notably, this constriction does not substantially recover, resulting in a persistently reduced pupil size throughout the remainder of the measurement period. This pattern suggests that images with higher visual complexity induce a strong initial pupillary constriction followed by sustained suppression of pupil size.

In contrast, Cluster 6, corresponding to the lowest spatial frequency, shows relatively minimal initial pupil constriction and a more pronounced recovery over time, leading to larger pupil sizes overall. Together, these contrasting patterns highlight the substantial influence of spatial frequency on the temporal dynamics of pupillary responses and underscore the importance of systematically controlling visual complexity when examining pupillary responses to emotional stimuli.

### 3.3 Arousal × SF interaction on valence classification

To directly assess the combined effects of emotional arousal and visual complexity on valence classification, models were trained under progressively more controlled conditions. When emotional arousal alone was controlled, classification performance showed modest improvements relative to the uncontrolled baseline, but remained limited overall (Table 6).

**TABLE 6:** Valence Classification Performance under Arousal‐Controlled Conditions.

Arousal	CV_folds	Model	Accuracy	Precision	Recall	Specificity	F1	AUC
Low	7	LightGBM	0.656 ± 0.063	0.695 ± 0.064	0.785 ± 0.129	0.470 ± 0.175	0.729 ± 0.066	0.679 ± 0.082
Mid	3	Bagging Classifier	0.559 ± 0.045	0.543 ± 0.066	0.446 ± 0.018	0.657 ± 0.096	0.487 ± 0.015	0.559 ± 0.030
High	7	Random Forest	0.654 ± 0.044	0.697 ± 0.027	0.816 ± 0.073	0.348 ± 0.090	0.751 ± 0.044	0.612 ± 0.070

*Note*: Performance of representative top‐performing models when emotional arousal is controlled but spatial frequency is not explicitly constrained. Values represent mean ± standard deviation across cross‐validation folds.

In contrast, when both emotional arousal and spatial frequency were jointly controlled, classification performance improved substantially across multiple arousal × spatial frequency combinations. For each condition, representative top‐performing configurations were selected based on mean AUC across cross‐validation folds (Table 7). These results demonstrate that jointly controlling emotional arousal and visual complexity yields more robust and reliable valence discrimination than controlling arousal alone.

**TABLE 7:** Valence classification performance under joint arousal and spatial frequency control.

Arousal	SF	CV_folds	k_neighbors	Model	Accuracy	Precision	Recall	Specificity	F1	AUC
Low	3	7	5	LightGBM	0.671 ± 0.140	0.683 ± 0.210	0.745 ± 0.170	0.610 ± 0.250	0.689 ± 0.140	0.772 ± 0.160
Low	4	5	5	Logistic Regression	0.789 ± 0.080	0.849 ± 0.090	0.838 ± 0.090	0.713 ± 0.180	0.838 ± 0.060	0.879 ± 0.100
Mid	2	7	5	Easy Ensemble	0.564 ± 0.100	0.539 ± 0.090	0.553 ± 0.130	0.57 ± 0.140	0.541 ± 0.110	0.586 ± 0.110
Mid	4	7	5	Random Forest	0.764 ± 0.070	0.132 ± 0.180	0.134 ± 0.180	0.901 ± 0.080	0.129 ± 0.170	0.547 ± 0.100
Mid	5	7	5	Easy Ensemble	0.586 ± 0.080	0.704 ± 0.060	0.668 ± 0.120	0.412 ± 0.140	0.681 ± 0.080	0.585 ± 0.110
High	1	7	5	Balanced Random Forest	0.744 ± 0.130	0.838 ± 0.080	0.817 ± 0.170	0.472 ± 0.320	0.818 ± 0.130	0.727 ± 0.170
High	3	7	5	SVM	0.695 ± 0.080	0.822 ± 0.12	0.756 ± 0.110	0.578 ± 0.300	0.776 ± 0.070	0.757 ± 0.140
High	4	5	5	Easy Ensemble	0.628 ± 0.100	0.432 ± 0.150	0.461 ± 0.200	0.702 ± 0.150	0.426 ± 0.130	0.679 ± 0.100
High	5	5	5	Bagging Classifier	0.687 ± 0.080	0.828 ± 0.090	0.739 ± 0.090	0.563 ± 0.200	0.776 ± 0.070	0.678 ± 0.110

*Note*: For each arousal × spatial frequency combination, the top‐performing configuration was selected based on mean AUC across cross‐validation folds. Values represent mean ± standard deviation.

To evaluate whether these performance gains were driven by synthetic oversampling, the same arousal × spatial frequency configurations were re‐evaluated without SMOTE (Table 8). Performance remained comparable across most conditions, with some configurations exhibiting similar or slightly higher accuracy and AUC values in the absence of oversampling. This pattern indicates that the observed improvements are not primarily attributable to synthetic data inflation, but rather reflect genuine structure in pupillary response patterns under controlled stimulus conditions.

**TABLE 8:** Valence classification performance under joint arousal and spatial frequency control (without SMOTE).

Arousal	SF	CV_folds	Model	Accuracy	Precision	Recall	Specificity	F1	AUC
Low	3	7	LightGBM	0.688 ± 0.140	0.697 ± 0.200	0.776 ± 0.150	0.626 ± 0.230	0.712 ± 0.130	0.748 ± 0.170
Low	4	5	Logistic Regression	0.759 ± 0.040	0.837 ± 0.09	0.804 ± 0.100	0.693 ± 0.19	0.811 ± 0.030	0.865 ± 0.1
Mid	2	7	Easy Ensemble	0.544 ± 0.080	0.519 ± 0.050	0.525 ± 0.090	0.554 ± 0.120	0.520 ± 0.060	0.584 ± 0.110
Mid	4	7	Random Forest	0.808 ± 0.030	0.000 ± 0.000	0.000 ± 0.000	0.985 ± 0.020	0.000 ± 0.000	0.453 ± 0.170
Mid	5	7	Easy Ensemble	0.584 ± 0.050	0.742 ± 0.040	0.588 ± 0.070	0.576 ± 0.080	0.654 ± 0.060	0.613 ± 0.080
High	1	7	Balanced Random Forest	0.711 ± 0.100	0.891 ± 0.090	0.715 ± 0.120	0.669 ± 0.270	0.785 ± 0.080	0.725 ± 0.190
High	3	7	SVM	0.714 ± 0.050	0.714 ± 0.050	1.000 ± 0.000	0.000 ± 0.000	0.832 ± 0.030	0.740 ± 0.110
High	4	5	Easy Ensemble	0.595 ± 0.090	0.407 ± 0.050	0.614 ± 0.270	0.581 ± 0.200	0.469 ± 0.11	0.712 ± 0.100
High	5	5	Bagging Classifier	0.650 ± 0.090	0.763 ± 0.05	0.777 ± 0.130	0.306 ± 0.130	0.763 ± 0.070	0.616 ± 0.150

*Note*: The same arousal × spatial frequency configurations reported in Table 7 are evaluated without oversampling to assess the robustness of the observed performance gains. Values represent mean ± standard deviation across cross‐validation folds.

**FIGURE 4:**
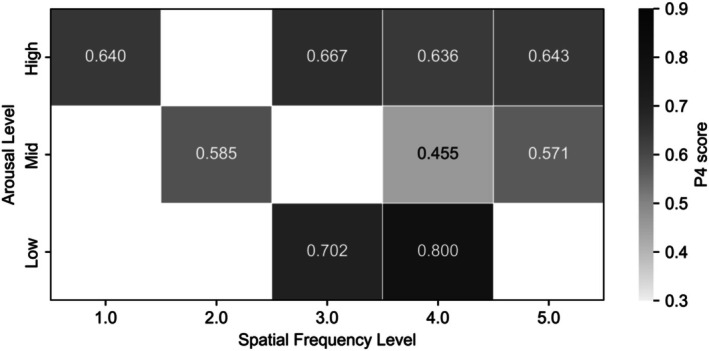
Heatmap of arousal × SF interaction. The heatmap shows classification accuracy (P4 score) across combinations of arousal level and spatial frequency. Values indicate mean accuracy for each condition. The black outline denotes the full arousal–spatial frequency condition space.

Finally, Figure 4 summarizes the interaction between emotional arousal and spatial frequency across the full condition space using the P4 score, a balanced classification metric. The heatmap reveals a non‐monotonic performance pattern across arousal levels, with higher performance observed under low and high arousal conditions and reduced performance under mid‐level arousal, further underscoring the importance of jointly considering emotional and perceptual factors in pupillometry‐based emotion classification.

**FIGURE 5:**
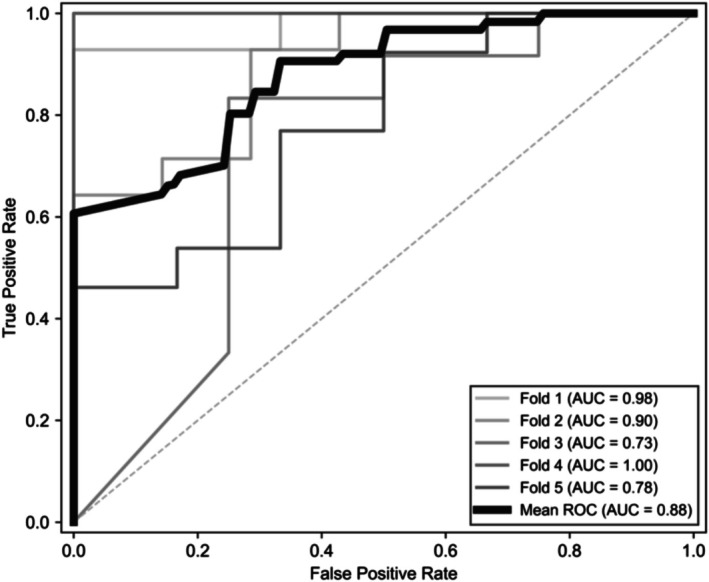
ROC curves generated from group 5‐fold cross‐validation. Thin gray lines represent ROC curves for individual folds, while the thick black line indicates the mean ROC curve across folds. The dashed diagonal line denotes chance‐level performance.

The ROC curve and AUC evaluation for a representative high‐performing configuration yielded a mean AUC of 0.88 (±0.10). Figure 5 shows the mean ROC curve along with the ROC curves for each cross‐validation fold. Notably, the ROC curve presented in this study exhibited a stepwise pattern in certain sections, which can be attributed to the inherent characteristics of binary classifiers and the limited sample size of the dataset. Specifically, when the size of test sets in each fold is small, changes in true positive rate and false positive rate tend to appear discontinuous, resulting in a stepwise appearance of the ROC curve (Fawcett, 2006).

### 3.4 Feature importance analysis

**FIGURE 6:**
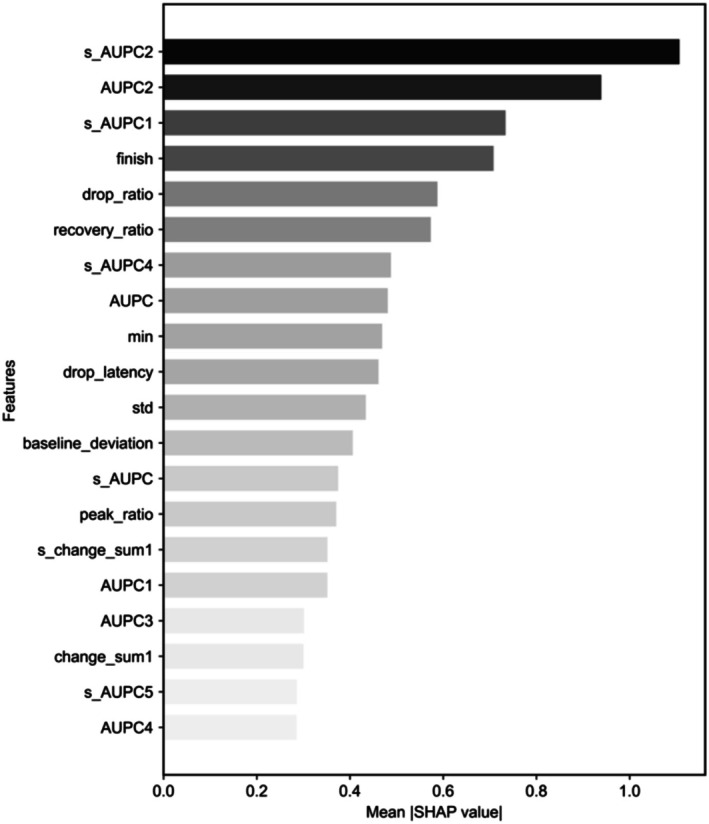
Feature importance generated by SHAP analysis (1). The bar plot shows the top 20 features ranked by mean absolute SHAP values for the logistic regression model trained under the low‐arousal, high‐spatial‐frequency condition. Feature labels are reported using the AUPC notation for consistency across analyses.

Figure 6 shows the results of the feature importance analysis, using SHAP values, to identify which specific pupillary response features most substantially contributed to classification performance. The features with the highest importance were related to the area under the pupil curve (AUPC). These results clearly indicate that overall pupil size characteristics played a crucial role in predicting emotional valence.

**FIGURE 7:**
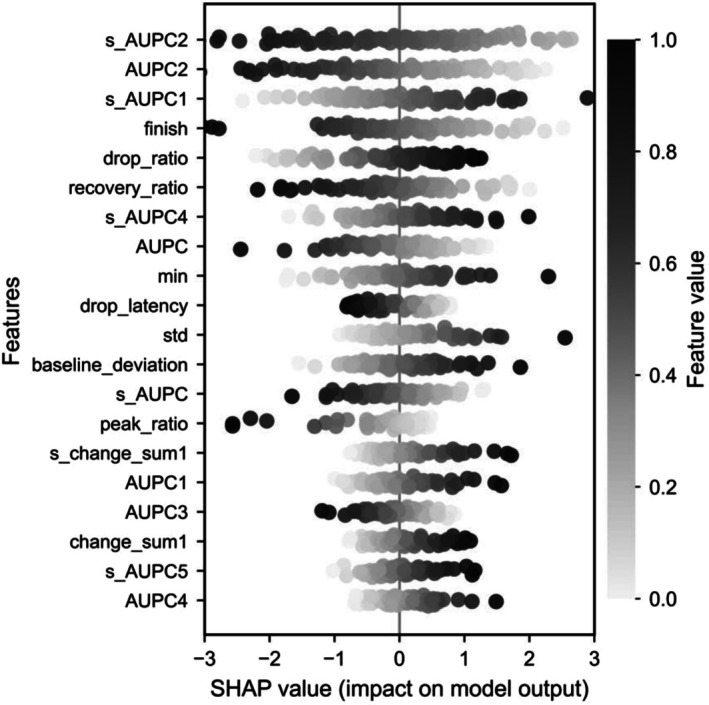
Feature importance generated by SHAP analysis (2). The dot plot shows the distribution of SHAP values for the same top features reported in Figure 6, illustrating both the magnitude and direction of feature contributions to the logistic regression model trained under the low‐arousal, high‐spatial‐frequency condition. Each point represents an individual sample, with color indicating the normalized feature value. Positive SHAP values indicate an increased likelihood of the positive valence class. All figures were created using Python3 with Matplotlib.

In Figure 7, the analysis of SHAP values revealed that positive emotional valence was primarily characterized by pupil response features, indicating overall larger pupil size and sustained dilation patterns. Specifically, higher values in total pupil dilation area (AUPC, and s_AUPC) and larger pupil dilation in intermediate time intervals (e.g., s_AUPC2, AUPC2, and AUPC3) were positively associated with positive valence. Furthermore, a larger pupil size at the final measurement point (finish), greater recovery of pupil size compared to the initial size (recovery_ratio), longer time to reach minimum pupil size (drop_latency), and a high ratio between peak and minimum pupil size (peak_ratio) were also indicative of positive valence. Together, these results indicate that positive emotional states are characterized by stable and sustained pupil dilation, delayed pupil constriction, and effective recovery to larger pupil sizes.

Conversely, negative emotional valence was predominantly associated with pupil response features indicating rapid constriction, smaller pupil sizes, and instability in pupil size changes. Specifically, higher values in features representing rapid and pronounced initial pupil constriction (drop_ratio and s_AUPC1) and smaller minimum pupil size (min) predicted negative valence. Moreover, features reflecting greater variability and instability in pupil size changes (std, baseline_deviation, and s_change_sum1) were positively associated with negative valence. Additionally, pronounced pupil size variations occurring in specific later intervals (s_AUPC5 and AUPC4) further predicted negative valence. Collectively, these findings suggest that negative emotional states are characterized by rapid initial pupil constriction, smaller overall pupil size, and more unstable, fluctuating patterns of pupil responses.

## 4 DISCUSSION

The present study revisited the potential of pupillary responses for emotional valence classification (positive vs. negative) using a machine‐learning framework, with a particular emphasis on the role of stimulus‐level confounding factors. Initial analyses conducted without explicit control of emotional arousal and spatial frequency yielded relatively modest classification performance (~57.1%). Importantly, this result should not be interpreted as evidence that pupillary responses lack valence‐related information. Rather, it aligns with the central motivation of the present study: when strong stimulus‐driven factors such as arousal and visual complexity are not controlled, these factors can dominate pupillary dynamics and obscure more subtle valence‐related signals. After explicitly controlling emotional arousal and spatial frequency, classification performance improved substantially (~81.9%), indicating that systematic control of stimulus properties is critical for enhancing the detectability and robustness of valence‐related pupillary patterns.

From a theoretical perspective, these findings help reconcile inconsistencies in prior literature regarding the relationship between pupil size and emotional valence (Bradley et al., 2008; Hess, 1965). Rather than reflecting a direct and universal mapping between valence and pupil dilation, pupillary responses appear to encode valence in a manner that is highly contingent on concurrent stimulus properties. In this context, spatial frequency—an often‐overlooked dimension of visual complexity—emerged as a key factor shaping pupillary dynamics. The present results suggest that previously reported null or inconsistent valence effects may, at least in part, stem from insufficient control of visual complexity and emotional arousal in earlier studies.

At the same time, the present findings should be interpreted with appropriate caution. One important limitation concerns the incomplete coverage of arousal × spatial frequency combinations within the stimulus set. Because stimuli were not systematically generated to populate all possible condition cells, some arousal × spatial frequency combinations were underrepresented or entirely absent. As a result, condition‐level performance differences should be interpreted conservatively. Sparse sampling can artificially accentuate apparent differences between conditions or mask variability that might emerge under more uniformly balanced experimental designs.

This limitation is especially relevant for low‐arousal conditions. Although classification performance under low‐arousal conditions was relatively high, this finding does not necessarily indicate stronger physiological differentiation in such states. Instead, it may reflect reduced within‐condition variability, residual visual influences, or sampling effects specific to the available stimulus set. Accordingly, interpretation in the present study is restricted to discrete condition‐level results rather than assuming smooth or continuous trends across arousal × spatial frequency space. Future studies employing systematically balanced stimulus designs will be necessary to robustly characterize interaction effects between emotional arousal and visual complexity.

Another limitation of the present study is the absence of participant‐reported valence and arousal ratings. Although arousal levels were defined using established IAPS‐based criteria, subjective emotional experiences were not directly assessed. Consequently, the present findings primarily reflect stimulus‐driven and physiological distinctions rather than individual subjective evaluations of emotional valence or arousal. While this limits direct inference about the relationship between subjective emotional experience and pupillary responses, it also enabled a focused examination of stimulus‐level and physiological factors under tightly controlled conditions. Future research incorporating participant‐specific affective ratings will be important for examining how subjective emotional experience interacts with pupillary dynamics at the individual level.

Finally, although group k‐fold cross‐validation and conservative oversampling strategies were employed to mitigate data leakage and class imbalance, the relatively small sample size remains a limitation of the present study. In this context, future work may further strengthen statistical inference by estimating empirical chance levels using permutation‐based testing, which would provide a data‐driven benchmark for assessing whether observed classification performance reliably exceeds chance expectations. Such approaches would complement the cross‐validation–based evaluation strategy adopted here, particularly in settings involving limited sample sizes and complex experimental designs. Future studies with larger and more diverse participant samples, including cross‐cultural and clinical populations, as well as the use of external validation datasets, will be necessary to further establish the generalizability of pupillometry‐based emotional valence classification. Extending this approach to dynamic stimuli, such as emotionally evocative video clips or standardized textual materials, may also help reduce residual visual confounds while increasing ecological validity.

Overall, the present study demonstrates that pupillary responses can carry meaningful information about emotional valence, but that such information becomes reliably detectable only when strong stimulus‐driven confounds are explicitly controlled. These findings underscore the importance of careful experimental design and nuanced interpretation when applying pupillometry to the study of emotion.

## 5 CONCLUSION

The present study demonstrates that pupil‐based emotional valence classification using machine‐learning approaches can be substantially improved when stimulus‐driven confounding factors are systematically controlled. In particular, explicitly controlling emotional arousal and spatial frequency led to a marked improvement in classification performance, increasing accuracy from 57.1% under uncontrolled conditions to 81.9% under controlled conditions. These findings underscore the critical importance of stimulus‐level control in pupillometry‐based emotional assessments, especially when subtle valence‐related signals are of interest.

By highlighting the role of visual complexity—operationalized here as spatial frequency—this study contributes to resolving inconsistencies in prior pupillometry research, in which emotional valence effects have often appeared weak or unreliable. Rather than indicating an absence of valence‐related information, such inconsistencies may reflect the dominance of uncontrolled stimulus‐driven factors that obscure pupillary dynamics.

From an applied perspective, the results support pupillometry as a potentially efficient and objective tool for emotional evaluation, provided that experimental design and stimulus properties are carefully controlled. Future research employing larger and more diverse samples, systematically balanced stimulus sets, and participant‐reported affective ratings will be essential for further validating and extending pupil‐based emotional valence classification systems across educational, clinical, and real‐world contexts.

## FUNDING INFORMATION

This work was supported by the Ministry of Education of the Republic of Korea and the National Research Foundation of Korea (NRF‐2024S1A5C3A01043667).

## CONFLICT OF INTEREST STATEMENT

The authors declare that they have no relevant financial or non‐financial interests to disclose.

## ETHICS STATEMENT

This study was approved by the Institutional Review Board (IRB) of Dongguk University (IRB approval number: DUIRB‐2024‐06‐20). All participants provided informed consent after being informed about the study objectives and procedures.

## Data Availability

The dataset analyzed in the current study is not publicly available due to ethical considerations related to IRB approval. However, anonymized data with all personally identifiable information removed can be provided by the corresponding author upon reasonable request and clear statement of purpose.
